# Data for glomeruli characterization in histopathological images

**DOI:** 10.1016/j.dib.2020.105314

**Published:** 2020-02-24

**Authors:** Gloria Bueno, Lucia Gonzalez-Lopez, Marcial Garcia-Rojo, Arvydas Laurinavicius, Oscar Deniz

**Affiliations:** a VISILAB, Universidad de Castilla-La Mancha, Ciudad Real, Spain; b Pathology Department, Hospital Universitario de Ciudad Real, Ciudad Real, Spain; c Pathology Department, Hospital Universitario Puerta del Mar, Cádiz, Spain; d Faculty of Medicine and National Center of Pathology, Vilnius, Lithuania

**Keywords:** Glomeruli identification, Normal glomerulus, Global sclerotic glomerulus, Whole slide image, Digital pathology

## Abstract

The data presented in this article is part of the whole slide imaging (WSI) datasets generated in European project AIDPATH 2 This data is also related to the research paper entitle “Glomerulosclerosis Identification in Whole Slide Images using Semantic Segmentation”, published in Computer Methods and Programs in Biomedicine Journal [1]. In that article, different methods based on deep learning for glomeruli segmentation and their classification into normal and sclerotic glomerulous are presented and discussed. The raw data used is described and provided here. In addition, the detected glomeruli are also provided as individual image files. These data will encourage research on artificial intelligence (AI) methods, create and compare fresh algorithms, and measure their usability in quantitative nephropathology.

**Table:** Specifications Table

Subject	*Pathology and Medical Technology*
Specific subject area	*Digital images in pathology. Annotated and classify whole slide image (WSI). Histopathology.*
Type of data	*Images, Whole Slide Images.*
How data were acquired	*Hardware: Whole Slide Digital Image Scanner (Leica Aperio ScanScope CS scanner) and extracted into an SVS file format.* *Software for annotation: Aperio ImageScope.* *Software for detection and extraction: VISILAB group software described in*Ref. [1]*.*
Data format	*Raw data: a) original images in SVS format* *Classified: Detected glomeruli to be used for classification in PNG format.*
Parameters for data collection	*Tissue samples were collected with a biopsy needle having an outer diameter between* 100 μm *and* 300 μm*. Afterwards, paraffin blocks were prepared using tissue sections of* 4 μm *and stained* using Periodic acid–Schiff *(PAS). Then, images at 20x magnification were selected.*
Description of data collection	*The tissue samples were scanned at 20x with a Leica Aperio ScanScope CS scanner.*
Data source location	*VISILAB, Universidad de Castilla-La Mancha, Ciudad Real, Spain*
Data accessibility	*Hosted by Mendeley at:*https://data.mendeley.com/datasets/k7nvtgn2x6/3
Related research article	Author's name: *Gloria Bueno, M. Milagro Fernandez-Carrobles, Lucia Gonzalez-Lopez, Oscar Deniz* Title: *Glomerulosclerosis Identification in Whole Slide Images using Semantic Segmentation* Journal: *Computer Methods and Programs in Biomedicine* https://doi.org/10.1016/j.cmpb.2019.105273

**Table:** 

**Value of the Data** • These data can be used for benchmarking to encourage further research on AI methods applied to digital pathology in nephrology. • The additional value of this data is that it has been acquired and evaluated by expert pathologists from different European countries. • All researches in digital pathology can benefit from these data, to test classification algorithms. And particularly for glomeruli identification in nephrology studies. • This data can be used for further development and new experiments in glomeruli classification with more classes, like focal glomeruli besides normal and sclerotic glomeruli. • The data will assist to provide further insights for nephropathologists, allowing to create novel diagnostic tools.

## 1 Data

The data is composed of two datasets:

1. DATASET_A: Raw data with 31 whole slide images (WSI) in SVS format. The size of the WSI range between 21651 × 10498 pixels and 49799 × 32359 pixles acquired at 20x. The images contain different types of glomeruli that were detected using the algorithms explained at [1]. The detected glomeruli are provided in DATASET_B.
2. DATASET_B: 2340 images with a single glomerulous, 1170 normal glomeruli and 1170 sclerosed glomeruli. All of them are in PNG format.

## 2 Experimental design, materials, and methods

The introduction of digital pathology to nephrology offers a platform to develop fresh methodologies and protocols for morphometric and computer-aided renal biopsies evaluation. Digital imaging application to pathology may provide significant advance for clinical studies and translational research. The international application of this new technology is driving new collaborative methods, providing unique opportunities but also challenges in education [2] and clinical practice [3]. One of the main challenges is the need for data acquired, evaluated and annotated to develop robust algorithms suitable for diagnosis [4].

Objective instruments to quantify and characterize glomeruli are needed for glomerulosclerosis assessment. The number of glomeruli is often used to determine the adequacy of a kidney biopsy and pathologists tend to underreport the number of glomeruli on a biopsy specimen [5].

The European project AIDPATH^2^ aware of the above-mentioned challenges carried out a data set of different whole slide images for breast and kidney tissue acquired by different European institutions under the same protocol. In this paper we supply some of the kidney tissue prepared at three different Pathology Department at Hospital Universitario de Ciudad Real (ES), Hospital Universitario Puerta del Mar (ES) and National Center of Pathology (LT) for glomerulosclerosis analysis.

Tissue samples were collected with a biopsy needle having an outer diameter between 100 μm and 300 μm. Afterwards, paraffin blocks were prepared using tissue sections of 4 μm and stained using PAS. Then, images at 20x magnification were selected. PAS stain is commonly used due to its efficiency dyeing polysaccharides, which are present in kidney tissue and in highlighting glomerular basement membranes [6,7]. A dataset of 47 kidney WSIs was obtained. Images at 20x magnification were selected since this magnification maintain image quality and information at the same as allows to obtain valuable results reducing computational time significantly.

Once WSIs at 20x magnification were collected, they were split into 2000 × 2000 pixels patches selecting only those which contained tissue. This set of patches was examined and labeled into three classes: i) Non-Glomerular structures: kidney tissue structures such as proximal and distal tubules, blood vessels, connective tissue stroma or inflammatory cells; ii) Normal Glomeruli characterized by thin glomerular capillary loops, a regular number of endothelial and mesangial cells. The aspect of glomerulus surrounding tubules is normal and iii) Sclerosed Glomeruli where the whole (or nearly the whole) glomerulus presents sclerosis.

The output of the previous steps was a dataset with a total of 1055 kidney tissue images, i.e., WSI subsamples with a size of 2000 × 2000 pixels. Glomeruli contours were annotated by pathologists using the Aperio ImageScope software.^3^ Then, per each subsample a gray mask was created containing: i) black pixels for the non-glomerular structures, ii) white pixels for the normal glomeruli and iii) gray pixels for the sclerosed glomeruli. As a result, 1245 glomerular structures were annotated, 303 of these were sclerosed glomeruli and 942 were normal glomeruli. These structures were extracted and data augmentation was performed onto these original images applying rotations of 90° and 270°, vertical flip and color transfer with 5 different references taken from the WSI provided at the Hospitals involved (see the associated publication for further details [1]). The purpose of data augmentation was twofold, 1st to augment the data as input of deep learning algorithms and 2nd to balance the number of normal and sclerosed glomeruli.

**Fig. 1:**
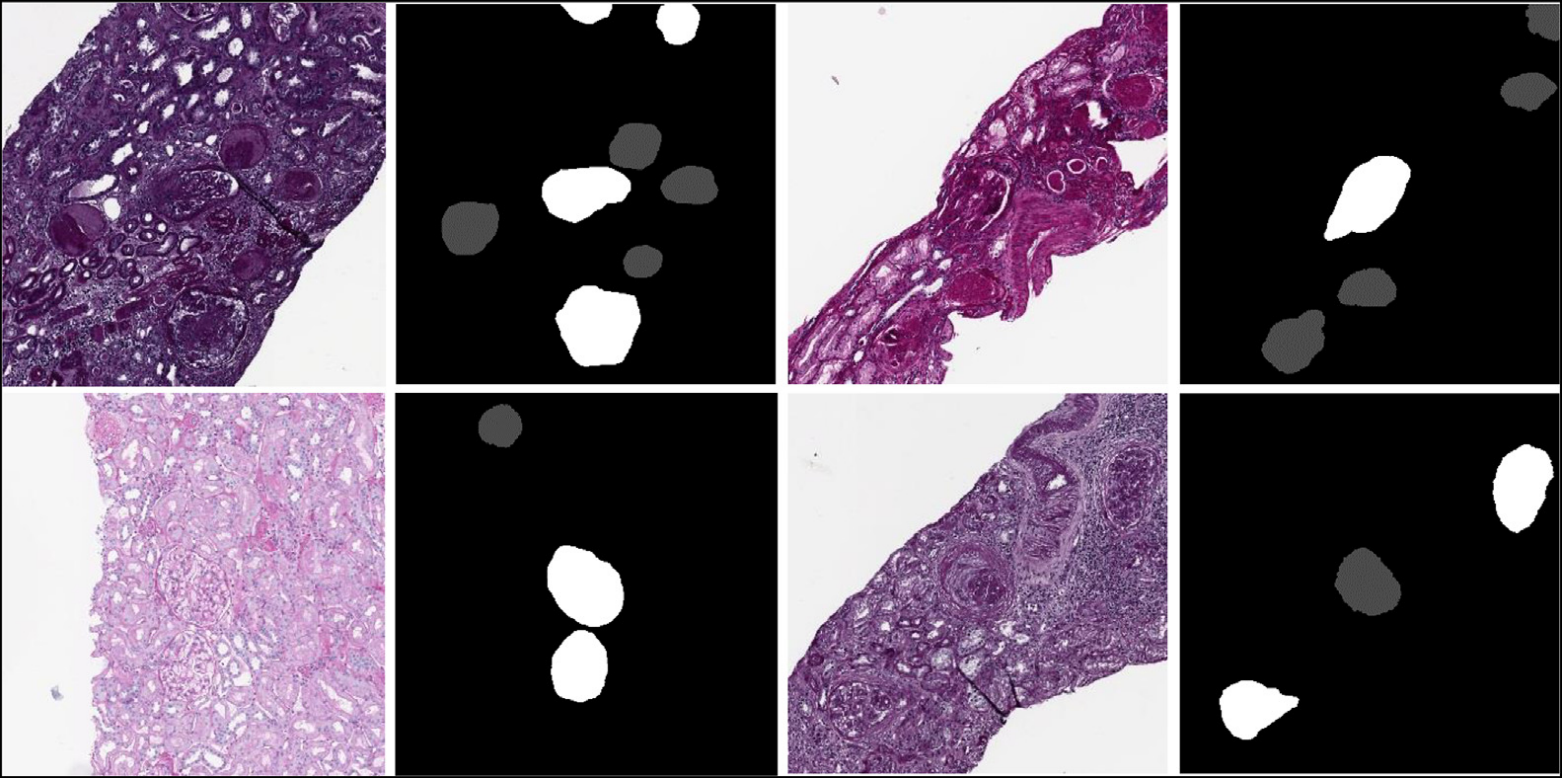
Samples of the DATASET_A, 1st an 3rd columns correspond to original images (raw data), 2nd and 4th columns are the masks of the segmented regions, that is the ground truth of the three classes considered: non-glomerular structures, normal glomeruli and sclerosed glomeruli. a) normal glomeruli, b) sclerosed glomeruli.

**Fig. 2:**
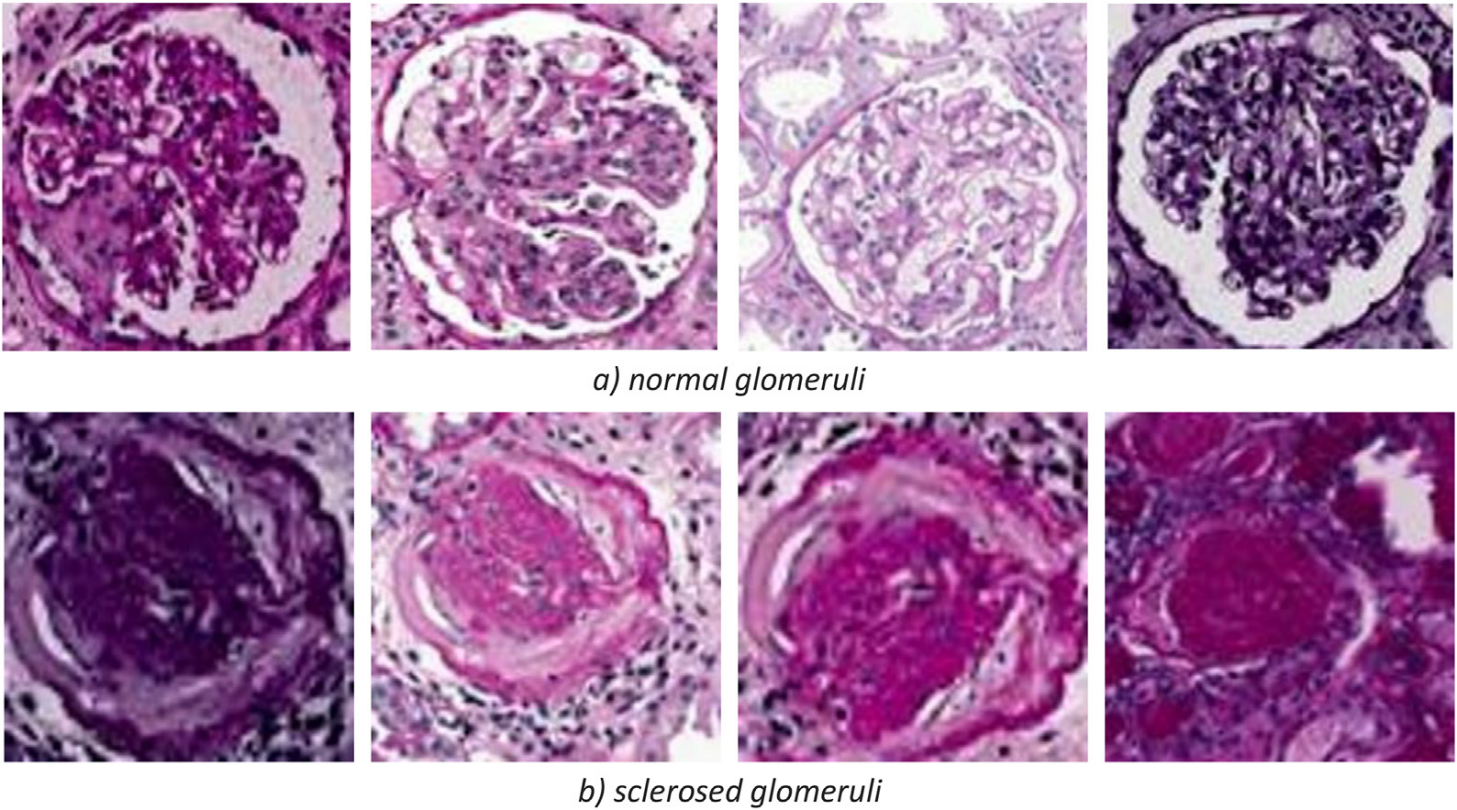
Samples of the DATASET_B provided for classification of two classes, normal and sclerosed glomeruli.

The raw data used (DATASET_A) together with the detected glomeruli (DATASET_B) are provided alongside this publication. Fig. 1, Fig. 2 illustrate patches of the raw data provided in this article and the detected glomeruli.

## Footnotes

3 https://www.leicabiosystems.com/digital-pathology/manage/aperio-imagescope/.
